# Characterizations of microRNAs involved in the molecular mechanisms underlying the therapeutic effects of noni (*Morinda citrifolia* L.) fruit juice on hyperuricemia in mice

**DOI:** 10.3389/fnut.2023.1121734

**Published:** 2023-06-22

**Authors:** Yue Liu, Xianjun Liu, Mengyuan Wang, Changwu Chen, Xiaohong Li, Zhiyong Liang, Yaming Shan, Yuhe Yin, Fengjie Sun, Zhandong Li, Hao Li

**Affiliations:** ^1^College of Biological and Food Engineering, Jilin Engineering Normal University, Changchun, China; ^2^School of Chemistry and Life Science, Changchun University of Technology, Changchun, China; ^3^Qingdao Haoda Marine Biotechnology Co., Ltd., Qingdao, China; ^4^National Engineering Laboratory for AIDS Vaccine, School of Life Sciences, Jilin University, Changchun, China; ^5^Key Laboratory for Molecular Enzymology and Engineering, Ministry of Education, School of Life Sciences, Jilin University, Changchun, China; ^6^School of Science and Technology, Georgia Gwinnett College, Lawrenceville, GA, United States

**Keywords:** hyperuricemia, mice, *Morinda citrifolia* fruit juice, microRNA, xanthine oxidase, uric acid, creatinine, blood urine nitrogen

## Abstract

**Background:**

Hyperuricemia is generally defined as the high level of serum uric acid and is well known as an important risk factor for the development of various medical disorders. However, the medicinal treatment of hyperuricemia is frequently associated with multiple side-effects.

**Methods:**

The therapeutic effect of noni (*Morinda citrifolia* L.) fruit juice on hyperuricemia and the underlying molecular mechanisms were investigated in mouse model of hyperuricemia induced by potassium oxonate using biochemical and high-throughput RNA sequencing analyses.

**Results:**

The levels of serum uric acid (UA) and xanthine oxidase (XOD) in mice treated with noni fruit juice were significantly decreased, suggesting that the noni fruit juice could alleviate hyperuricemia by inhibiting the XOD activity and reducing the level of serum UA. The contents of both serum creatinine and blood urine nitrogen of the noni fruit juice group were significantly lower than those of the model group, suggesting that noni fruit juice promoted the excretion of UA without causing deleterious effect on the renal functions in mice. The differentially expressed microRNAs involved in the pathogenesis of hyperuricemia in mice were identified by RNA sequencing with their target genes further annotated based on both Gene Ontology and Kyoto Encyclopedia of Genes and Genomes databases to explore the metabolic pathways and molecular mechanisms underlying the therapeutic effect on hyperuricemia by noni fruit juice.

**Conclusion:**

Our study provided strong experimental evidence to support the further investigations of the potential application of noni fruit juice in the treatment of hyperuricemia.

## 1. Introduction

Hyperuricemia in either males or females is generally defined based on the solubility of uric acid (UA), i.e., the serum UA concentration over 7.0 mg/dL or 416 mmol/L (1), which is caused by either excessive UA production (2) and/or decreased excretion in kidney and intestine, ultimately leading to excessive increase of serum UA concentration (3, 4). Furthermore, hyperuricemia is well known as an important risk factor for the development of various medical disorders, e.g., hypertension, diabetes, cardiovascular complication, metabolic syndrome, atherosclerosis, stroke, gout, and kidney diseases (5–7), even though it is recommended that the asymptomatic hyperuricemia may not be treated (8). Unfortunately, the commonly applied clinical treatments of hyperuricemia, i.e., the urate-lowering therapies, including the use of xanthine oxidase (XOD) inhibitors and other uricosuric drugs, often cause severe side effects (9). According to the American College of Rheumatology Guidelines for Management of Gout Part I in 2012, the first-line treatment strategy for lowering the content of UA is to use XOD inhibitor, i.e., allopurinol (10), while the application of allopurinol is frequently associated with multiple side-effects, including increased toxicity with low glomerular filtration rate resulting in accelerated risk of allopurinol hypersensitivity syndrome, hepatotoxicity, and Stevens-Johnson syndrome (11). Therefore, it is clinically urgent to develop effective and safe drugs in the therapeutic treatment of hyperuricemia. Recently, probiotics have been revealed with potential treatment and prevention of hyperuricemia. For example, the serum UA levels are significantly decreased in mice by the treatments of *Lactobacillus fermentum* F40-4 (12) and *Lactiplantibacillus pentosus* P2020 (13). In addition, oral delivery of nanoparticles with uricase has shown protective effects on mice with hyperuricemia (14).

Notably, plants have a long history of being widely used in traditional medicines for the treatment of various diseases (15). Medicinal plants have also played an important role in drug discovery, development, and production (16). In particular, the plants of *Morinda citrifolia* L., popularly known as noni, are one of the commonly used traditional medicinal plants discovered by Polynesian ancestors and have been used in Polynesia and almost worldwide for over 2000 years (17). Traditionally, noni has been used in the treatment of various diseases and medical disorders, including cancer, infection, cold, flu, diabetes, hypertension, arthritis, gastric ulcer, sprain, depression, senility, muscle ache, and pain (18). Furthermore, the modern pharmacological studies have revealed the anti-inflammatory, antioxidant, hepatoprotective, and immunomodulatory effects of noni (19). In our previous studies, the noni fruit juice showed significantly therapeutic effect on acute gouty arthritis, which is closely related to hyperuricemia, with a group of differentially expressed microRNAs (miRNAs) involved in the pathogenesis of acute gouty arthritis in mice identified using the high-throughput RNA sequencing (RNA-Seq) (20). Furthermore, studies have shown that the inhibitory effect of noni fruit juice on the enzymatic activities of XOD could explain the underlying mechanisms ameliorating gout and gout-like diseases (21). To date, the molecular mechanisms regulating the therapeutic effects of noni fruit juice on the treatment of hyperuricemia are unclear. Therefore, it is necessary to further investigate the biochemical and molecular associations between noni fruit juice and hyperuricemia in order to provide the experimental evidence to support the potential application of noni fruit juice in the clinical treatment of hyperuricemia.

The molecular mechanisms underlying hyperuricemia have been extensively investigated with various genetic factors and metabolic pathways involved in the occurrence and treatment of hyperuricemia identified. For example, a group of miRNAs involved in hyperuricemia have been identified (22). The miRNAs are small non-coding RNAs, with an average of 22 nucleotides in length and widely found in various organisms (23), functioning to regulate the stability of target messenger RNAs (mRNAs) by selectively binding to specific sites. With the rapid advancements in the understanding of the regulatory functions of miRNAs involved in various biological activities, it is highly expected that the targeted studies focusing on the exploration of the relationships between miRNAs and various types of human diseases would significantly enhance the identification and further development of novel therapeutic targets in the treatments of these medical disorders. For example, the increasing evidence has suggested that the development of hyperuricemia and gout is regulated at the post transcriptional level with strong involvement of miRNAs (24). Furthermore, studies have revealed abnormal expressions of miRNAs involved in hyperuricemia and the association between serum UA concentration and altered expressions of miRNAs (25). In particular, miRNAs could regulate the expression of urate transporter genes, e.g., miR-34a regulates the mRNA of the *solute carrier family 22 member 12* (*SLC22A12*) gene to ultimately repress the uric acid transporter 1 (URAT1) expression in the animal model of hyperuricemia (26). These studies strongly indicate that miRNAs could be potentially used as the therapeutic biomarkers for the clinical prevention and treatment of hyperuricemia.

The objective of this study was to investigate the effect of noni fruit juice on the alleviation of hyperuricemia and the underlying molecular mechanisms in mouse model of hyperuricemia induced by the uricase inhibitor potassium oxonate (PO) using biochemical and high-throughput sequencing analyses. The levels of a group of four hyperuricemia related biochemical factors, i.e., serum UA, XOD, creatinine (Cr), and blood urine nitrogen (BUN), were detected in the mouse model of hyperuricemia treated with either noni fruit juice or allopurinol. The RNA-Seq was used to identify the differentially expressed miRNAs involved in the pharmacological regulation of hyperuricemia by noni fruit juice. The target genes of these differentially expressed miRNAs were further annotated using the both Gene Ontology (GO) and Kyoto Encyclopedia of Genes and Genomes (KEGG) databases to explore the metabolic pathways and molecular mechanisms underlying the therapeutic effect on hyperuricemia by noni fruit juice. This study strongly demonstrated the regulatory functions of miRNAs in the treatment of hyperuricemia in mice by noni fruit juice, providing strong experimental evidence to support the further investigations of the biochemical and molecular associations between noni fruit juice and hyperuricemia as well as the potential application of noni fruit juice in the clinical treatment of hyperuricemia.

## 2. Materials and methods

### 2.1. Production of noni fruit juice

Fresh and mature noni fruits were purchased from the Fiji Pacific Noni Biotechnology Co., Ltd. (Hainan, China). These fruit materials were locally collected (Nadi, Fiji) and kept frozen at −20°C until further use. The frozen fruits were processed as previously reported (20). Briefly, the frozen fruits were first thawed at room temperature and then rinsed using sterile water. After being air dried, the fruits were cut into small pieces each of ~5 mm in thickness and crushed by a fruit crusher to make the fruit pulp, which was heated to ~25°C, added with pectinase (0.225 g/L) under constant stirring, and then inoculated with *Lactobacillus plantarum* (1%) to incubate for 4 h in an incubator (Shanghai Yiheng Scientific Instrument Co., Ltd., Shanghai, China) with constant temperature at 40°C. Then, the 20-mesh filter cloth was used to filter the fruit pulp, which was fermented for 20–30 d at 38°C. Finally, the supernatant was collected with a siphon, filtered, sterilized for 20 min at 80–82°C, and aseptically collected as the fermented noni fruit juice used in this study.

### 2.2. Animal treatments and induction of hyperuricemia in mice

A total of 60 male Kunming mice with an average body weight of 20 ± 2 g and the animal certificate number of SCXK (Liao)-2015–0001 were purchased from Liaoning Changsheng Biotechnology Co., Ltd., Benxi, China. The selection of male animals was based on the general knowledge that the male mice were prone to developing hyperuricemia than female mice. During the week of adaptive feeding, these 60 mice were randomly and evenly separated into six treatment groups as follows: the normal control group contained the mice treated with regular food and water, the model group treated with uricase inhibitor PO, the positive control group treated with both PO (250 mg kg^−1^; MedChemExpress, Shanghai, China) and allopurinol (5 mg kg^−1^; Shanghai Yuanye Bio-Technology Co., Ltd., Shanghai, China), and three noni fruit juice groups treated with both PO and noni fruit juice of 3.3 mL kg^−1^ d^−1^ (low-dose), 6.6 mL kg^−1^ d^−1^ (medium-dose), and 13.2 mL kg^−1^ d^−1^ (high-dose), respectively. The selection of these three dose levels was based on the results of our pre-experiments showing significant variations in different groups of mice. The mice were treated in compliance with the principles of laboratory animal care and the guide for the care and use of laboratory animals approved by the Ethics Committee of Jilin University (approval # 2018SY0602). All mice were caged and fed *ad libitum*, under natural sunlight and constant temperature and humidity, and were regularly cleaned and disinfected. The mice in the noni fruit juice group with the optimal effects on hyperuricemia related biochemical factors were selected for further RNA-Seq analysis.

To make the mouse model of hyperuricemia, the treatment with PO was used to induce hyperuricemia in mice based on the previous study (27). Except for the normal control group, each mouse in other five treatment groups was orally administered with PO at 8 am for 7 d to induce hyperuricemia. In 1 h, the mice in the noni fruit juice and the allopurinol groups were orally given noni fruit juice and allopurinol, respectively, for 7 d. The successful establishment of mouse model of hyperuricemia was determined by the significantly elevated level of serum UA (*p* < 0.0001) compared with the normal control group. On day 7, 1 h after the treatments, the mice were euthanized by inhalation of carbon dioxide gas.

### 2.3. Biochemical and statistical analyses

The whole blood sample was collected from each mouse 1 h after the 7th administration of required treatments, coagulated at room temperature for about 1 h, and then centrifuged for 5 min at 3000 rpm/min to obtain the serum. The contents of serum UA (1), XOD (9), Cr (28), and BUN (29) in the serum of each mouse were measured by the specific detection kits purchased from Nanjing Jiancheng Biotechnology Co., Ltd. (Nanjing, China) according to the manufacturer’s instructions.

All data were expressed as the mean ± standard error of the mean. All statistical analyses were performed using the one-way analysis of variance (ANOVA) to determine the level of significance based on *p* < 0.05.

### 2.4. High-throughput RNA sequencing and microRNA analyses

In order to further explore the molecular mechanisms regulating the effect of noni fruit juice and allopurinol on reducing the level of serum UA in mice with hyperuricemia, each representative serum sample was randomly selected for RNA sequencing. A total of 200 μL serum sample were added with 1 mL QIAzol Lysis Reagent (QIAGEN, Germany) in the sample tube, vortexed, and incubated at room temperature for 5 min. Then, the mixed sample was added with a total of 200 μL chloroform/isoamyl alcohol (24,1; v:v) solution, vortexed for 15 s, incubated at room temperature for 3 min, and then centrifuged for 8 min at 12,000 × *g* and 4°C. The supernatant was absorbed and added with anhydrous ethanol twice the volume of the supernatant, mixed well, and purified through the column. The sample was washed once with 700 μL buffer RWT and twice with 500 μL buffer RPE, and centrifuged for 2 min at room temperature and 12,000 × *g*, with the purification column moved to a new collection tube and added with 20 μL RNA-free water, incubated at room temperature for 1 min, and centrifuged for 2 min at room temperature and 12,000 × *g* to elute RNA, which was detected with Agilent 2100 Bioanalyzer (Agilent Technologies Co., Ltd., Beijing, China). Nanodrop was used to detect the salt ion pollution in the RNA samples.

The small RNA library was constructed as previously reported (20). The RNA-Seq was performed by combinatorial Probe-Anchor Synthesis (cPAs) to obtain the miRNAs by next generation sequencing platform BGISEQ-500 (BGI, Shenzhen, China) (30, 31). The raw data were filtered to remove tags of low quality, with 5′ primer contaminant, without 3′ primer, without insertion, with poly A, and shorter than 18 nt, to obtain the clean tags, which were mapped to the reference genome and the miRbase database (22nd edition) using Bowtie2 (32) and mapped to Rfam using the function “cmsearch” (33). Novel miRNAs were predicted using miRDeep2 (34). The expression levels of miRNAs were calculated by the transcripts per million kilobase transcript (TPM) method (35), which eliminated the effects of sequencing variations. The TPM method was performed using the following formula: TPM = (C*10^6^)/N, where C represented the count of miRNAs in a sample and N the total number of reads mapped to the reference genome. Based on the detection method of differentially expressed genes (DEGs) as previously reported (36), a strict in-house algorithm was developed to screen the DEGs of miRNAs between two samples with the multiple hypothesis test corrections set up (37) to determine the domain value of p by controlling the false discovery rate (FDR) (38).

Based on the default parameters, both MiRanda (39) and TargetScan (40) were used to predict the target genes of the differentially expressed miRNAs. The functional annotation and enrichment analyses of the target genes were further performed based on Gene Ontology (GO)^1^ and Kyoto Encyclopedia of Genes and Genomes (KEGG)^2^ databases, respectively. GO database is an international standardized gene function classification system, providing a set of dynamically updated standard vocabulary to comprehensively describe the attributes of genes and gene products in organisms, categorized into three groups of genes, i.e., molecular function, cellular component, and biological process, respectively. GO annotation was performed to identify the functional GO terms that were significantly enriched in the target genes corresponding to the differentially expressed miRNAs compared with the genomic background, ultimately detecting the biological functions that were significantly related to the target genes corresponding to the differentially expressed miRNAs. First, all target genes corresponding to differentially expressed miRNAs were directed to the GO database to calculate the number of genes of each GO term, and then the hypergeometric test was performed to identify the GO entries that were significantly enriched in the target genes corresponding to the differentially expressed miRNAs compared with the whole genome background. Based on GO::Termfinder^3^, a strict in-house algorithm was developed to perform the GO annotation analysis with the calculated *p*-value corrected by Bonferroni (37). The corrected value of *p* ≤ 0.05 was used as the threshold and the GO term meeting this condition was defined as the GO term significantly enriched in the target gene corresponding to the differentially expressed miRNAs. The enrichment analysis of metabolic pathways based on KEGG database was performed to investigate the biological function of targe genes of the differentially expressed miRNAs (41) and to identify the metabolic pathways that were significantly enriched by the target genes of differentially expressed miRNAs compared with the whole genome background based on *Q*-value ≤ 0.05.

## 3. Results

### 3.1. Effects of noni fruit juice on the levels of serum uric acid, xanthine oxidase, creatinine, and blood urine nitrogen in mice

**Figure 1:**
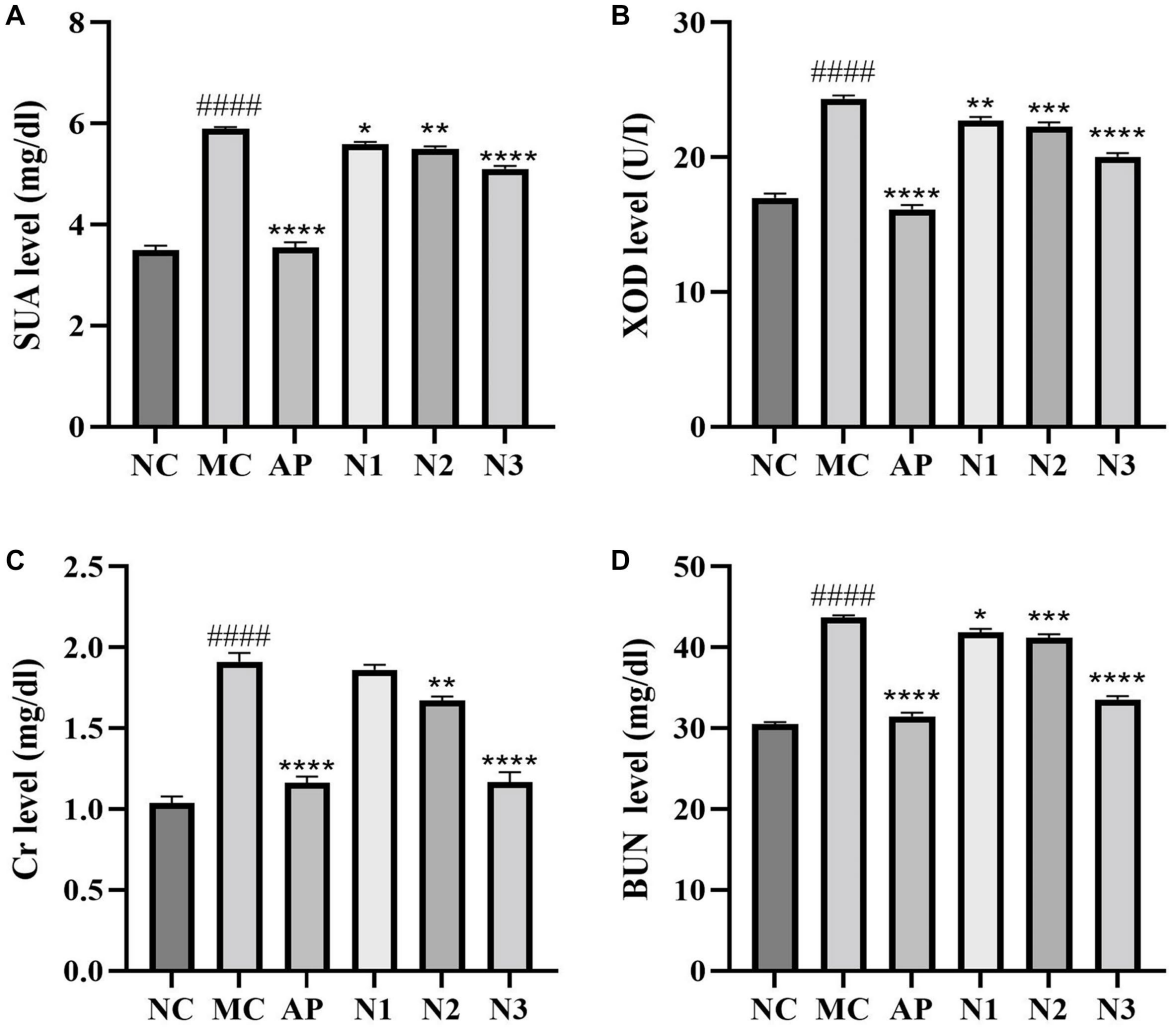
Effects of noni fruit juice on the contents of **(A)** serum uric acid (SUA), **(B)** xanthine oxidase (XOD), **(C)** creatinine (Cr), and **(D)** blood urine nitrogen (BUN) in six groups of mice, i.e., the normal control (NC), the model (MC), the positive control treated with allopurinol (AP), and low-dose (N1), medium-dose (N2), and high-dose (N3) noni fruit juice groups. Significant differences are determined based on *p* < 0.0001 (^####^) compared with the normal control group and *p* < 0.05 (*), *p* < 0.01 (**), *p* < 0.001 (***), and *p* < 0.0001 (****), compared with the model group, respectively.

Compared with the normal control group, the serum UA level of mice in the model group was significantly increased with the treatment of PO (*p* < 0.0001), indicating that the mouse model of hyperuricemia was successfully established (Figure 1A). Compared with the model group, the levels of serum UA in the allopurinol group (i.e., the positive control; *p* < 0.0001) and the noni fruit juice groups were significantly decreased (*p* < 0.05, *p* < 0.001, and *p* < 0.0001 for low-, medium-, and high-dose of noni fruit juice groups, respectively). These results revealed the therapeutic effect of noni fruit juice on hyperuricemia in mice in a dose-dependent manner. Compared with the normal control group, the content of XOD in mice of the model group was significantly increased with the treatment of PO (*p* < 0.0001; Figure 1B). Compared with the model group, the contents of XOD in both allopurinol (*p* < 0.0001) and noni fruit juice groups (*p* < 0.01, *p* < 0.001, and *p* < 0.0001 for low-, medium-, and high-dose noni fruit juice groups, respectively) were significantly reduced. These results showed that noni fruit juice could effectively reduce the enzymatic activity of XOD with curative effect on inhibiting the UA synthesis. To evaluate the changes in renal function of mice treated with noni fruit juice, the contents of serum Cr and BUN were measured in mice of hyperuricemia. The results showed that noni fruit juice could effectively alleviate the renal injury in mice of hyperuricemia. Compared with the normal control group, the content of serum Cr in the model group was significantly increased (*p* < 0.0001), whereas the contents of serum Cr in mice of both the allopurinol (*p* < 0.0001) and the noni fruit juice groups (*p* < 0.01 and *p* < 0.001 for medium- and high-dose noni fruit juice groups, respectively) were significantly lower than that of the model group (Figure 1C), though no significant difference was revealed in the Cr content between the low-dose noni fruit juice group and the model group of mice. Similar patterns were revealed in the changes of the contents of BUN (Figure 1D), i.e., compared with the normal control group, the contents of BUN in mice of the model group were significantly increased (*p* < 0.0001), whereas the contents of BUN in mice of both the allopurinol (*p* < 0.0001) and noni fruit juice groups (*p* < 0.05, *p* < 0.001, and *p* < 0.0001 for low-, medium-, and high-dose noni fruit juice groups, respectively) were significantly lower than that of the model group. These results suggested that the noni fruit juice did not cause deleterious impact on the renal function in mice with hyperuricemia. Due to its optimal effects on these biochemical indices, the mice in the high-dose noni fruit juice group were used for further RNA-Seq analysis.

### 3.2. RNA sequencing

#### 3.2.1. Statistics of microRNAs

In order to further explore the molecular mechanisms regulating the effect of noni fruit juice on the contents of hyperuricemia related biochemical indices in mice of hyperuricemia, the miRNAs of different groups of mice were sequenced using the BGISEQ-500 sequencing technology. The raw sequencing data were deposited in the Sequence Read Archive (SRA) of the National Center for Biotechnology Information (NCBI)^4^ database under the BioProjects PRJNA910471 and PRJNA719968. The raw data were filtered to obtain the clean tags, which were annotated using miRBase and Rfam databases to identify the known miRNAs, with the remaining unknown miRNAs used to predict novel miRNAs (Table 1 and Supplementary Table S1).

**Table 1:** Summary of microRNA sequencing data obtained from four groups of mice based on the BGISEQ-500 sequencing technology.

Sample	Raw tag	Clean tag (%)	Mapped tag (%)	Known miRNA	Novel miRNA
Normal control group	29,268,292	23,957,783 (81.86)	21,749,074 (90.78)	271	2,146
Model group	33,333,333	24,026,953 (72.08)	21,562,608 (89.74)	248	1,887
Noni fruit juice group	28,679,191	12,187,451 (42.5)	11,301,855 (92.73)	201	4,289
Allopurinol group	28,571,428	23,892,444 (83.62)	20,602,381 (86.23)	163	2,134

The percentage of clean tag is calculated as (clean tag counts/raw tag counts) × 100%. The percentage of mapped tag is calculated as (mapped tag counts/clean tag counts) × 100%.

#### 3.2.2. Differentially expressed microRNAs

**Figure 2:**
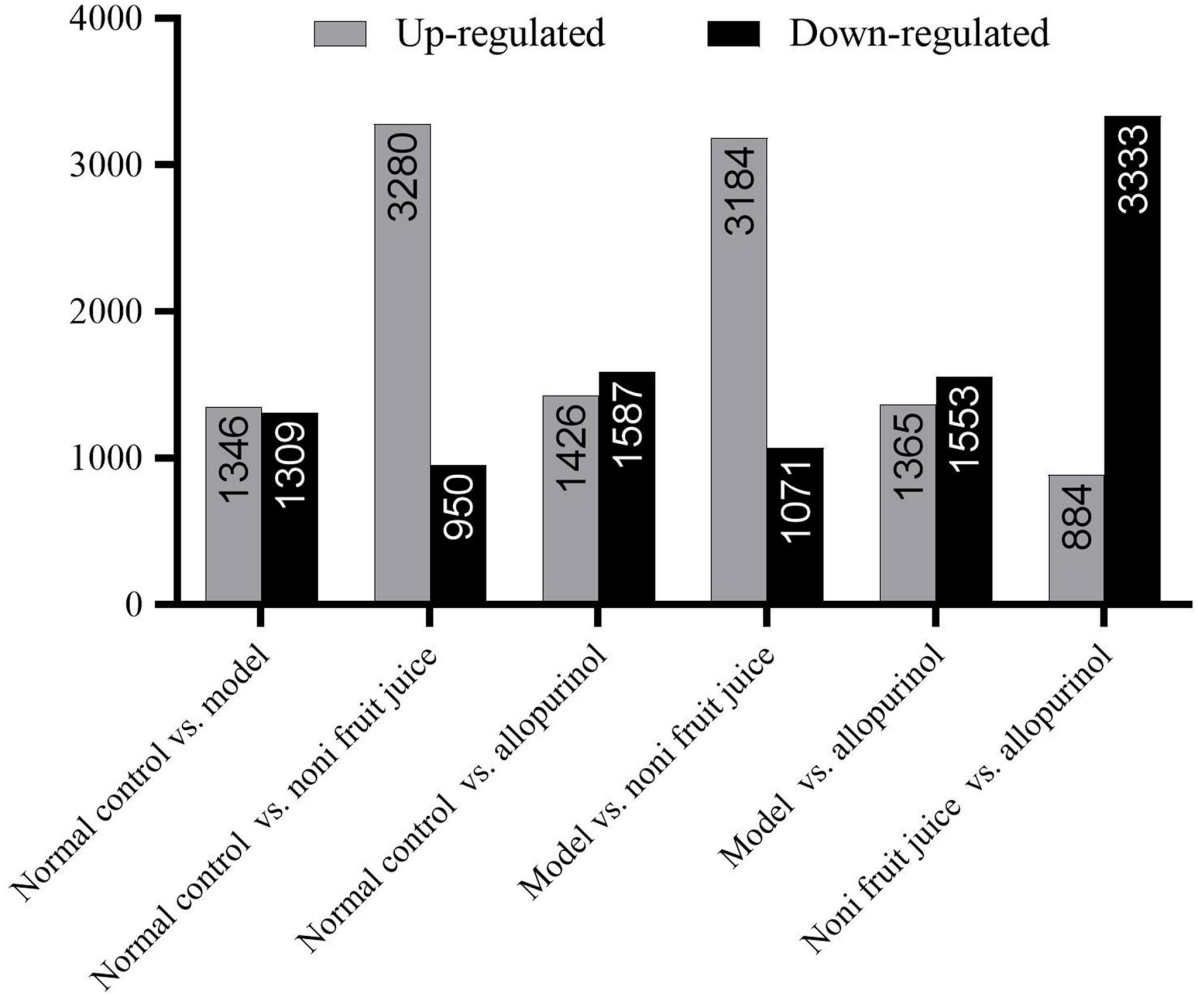
Summary of the differentially expressed microRNAs (up-regulated and down-regulated) identified in six pairwise comparisons of the four groups of mice, i.e., the normal control, the model, the noni fruit juice, and the allopurinol groups.

Differential expression analysis of miRNAs was performed using Expdiff with the PossionDis method based on FDR ≤ 0.001 and the absolute value of Log2Ratio(Fold Change) ≥ 1 as the default thresholds to determine the significance of expression variation and identify the differentially expressed microRNAs between samples (Figure 2 and Supplementary Table S2). The results showed that the largest numbers of differentially expressed miRNAs were detected in the noni fruit juice group compared with the other three groups of mice, i.e., a total of 3,280 and 3,184 up-regulated miRNAs were revealed in the pairwise comparisons of the normal control group vs. noni fruit juice group and the model group vs. noni fruit juice group, respectively, while a total of 3,333 down-regulated miRNAs were detected in the pairwise comparison of the noni fruit juice group vs. allopurinol group. These results evidently revealed the significant effect of noni fruit juice on mice with hyperuricemia. Further studies are needed to explore the explicit functions of these differentially expressed miRNAs in the development and treatment of hyperuricemia in mice.

#### 3.2.3. Prediction of the target genes of the differentially expressed microRNAs

**Figure 3:**
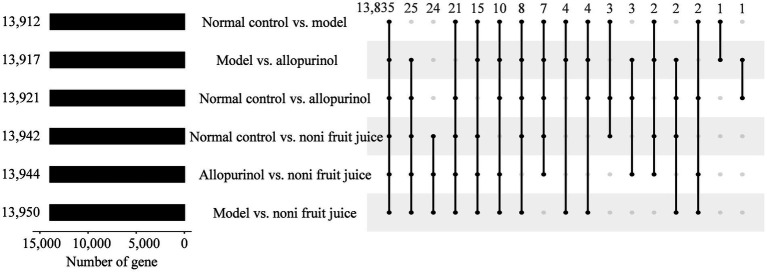
Target genes of differentially expressed microRNAs predicted by both TargetScan and miRanda in the six pairwise comparisons of the four groups of mice, i.e., the normal control, the model, the allopurinol, and the noni fruit juice groups. Numbers on the left indicate the total number of genes identified in the six pairwise comparisons; numbers on the top indicate the number of genes shared (i.e., intersection values) among the pairwise comparisons indicated by dots and lines.

To further determine the genes involved in the molecular mechanisms regulating the effect of noni fruit juice on the contents of hyperuricemia related biochemical indices in mice, the target genes of differentially expressed miRNAs were predicted using both TargetScan and miRanda (Figure 3 and Supplementary Table S3). A total of 13,912, 13,942, 13,921, 13,950, 13,917, and 13,944 target genes were detected in the six pairwise comparisons of four groups of mice, i.e., the normal control vs. model groups, the normal control vs. noni fruit juice groups, the normal control vs. allopurinol groups, the model group vs. the noni fruit juice groups, the model vs. allopurinol groups, and the allopurinol vs. the noni fruit juice groups (Supplementary Tables S4–S9), respectively.

#### 3.2.4. GO functional annotation of target genes of differentially expressed microRNAs

**Figure 4:**
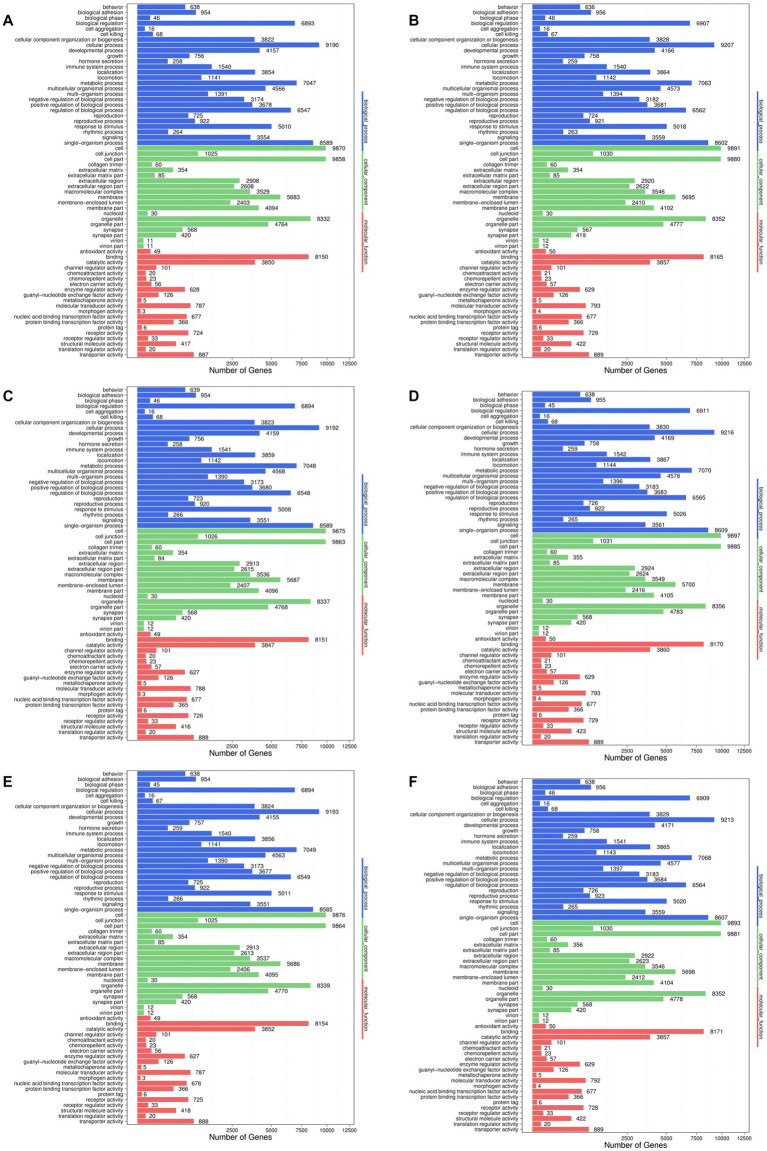
Functional annotations of the target genes of differentially expressed microRNAs based on the Gene Ontology (GO) database identified in the pairwise comparisons of the four groups of mice, i.e., the normal control group vs. model group **(A)**, the normal control group vs. noni fruit juice group **(B)**, the normal control group vs. allopurinol group **(C)**, the model group vs. noni fruit juice group **(D)**, the model group vs. allopurinol group **(E)**, and the allopurinol group vs. noni fruit juice group **(F)**. The GO terms are grouped in to three categories, i.e., biological process, cellular components, and molecular function.

In order to further explore the effect of noni fruit juice on hyperuricemia in mice at the genomic level, the target genes of the differentially expressed miRNAs were further annotated based on the GO database (Figure 4). The results showed that the most target genes of the differentially expressed miRNA were annotated in the category of biological process, followed by the categories of cellular component and molecular function, of the GO database. A total of 26 GO functional groups were revealed in the category of biological processes, e.g., the GO terms in cellular process, single-organism process, and metabolic process. In the category of cellular component, a total of 19 GO terms were annotated, with most target genes annotated in the GO terms of cell and cell part, followed by organelle. The category of molecular mechanism contained a total of 20 GO functional groups, with the most target genes annotated in the GO term of binding, followed by catalytic activity. Subsequently, these most annotated GO functional groups were further explored to investigate the molecular mechanisms underlying the effect of noni fruit juice on lowering the content of serum UA in mice with hyperuricemia.

It is commonly known that UA is the final product of the purine metabolism in human, while hyperuricemia is mainly caused by either excessive UA produced by purine degradation pathway or insufficient excretion of UA by interfering with the UA uptake transporters and secretory transporters. Therefore, as one of the main pathways involved in the pathology of hyperuricemia, the urate biosynthetic process, urate metabolic process, and urate transport were further characterized based on GO annotation of target genes of differentially expressed miRNAs identified in six pairwise comparisons of the four groups of mice (Table 2 and Supplementary Table S10). The results showed that in the biological process category of GO database, many up-regulated and down-regulated miRNAs were involved in the positive and negative regulations of the metabolic pathways of urate biosynthetic process, urate metabolic process, and urate transport. These results strongly indicated that the urate biosynthetic process, urate metabolic process, and urate transport pathways were involved in the regulatory function of noni fruit juice in mice with hyperuricemia, while these target genes of the differentially expressed miRNAs annotated in these GO terms were probably involved in the urate biosynthetic process, urate metabolic process, and urate transport. The known and novel miRNAs involved in the urate biosynthetic process, urate metabolic process, and urate transport were summarized in the pairwise comparisons of the four groups of mice (Table 3 and Supplementary Table S10). Further studies are needed to investigate the explicit regulatory functions of these miRNAs in the occurrence of hyperuricemia and the molecular mechanisms regulating the alleviation effect of noni fruit juice on hyperuricemia in mice.

**Table 2:** Target genes and the expression patterns of the differentially expressed microRNAs involved in the metabolism of uric acid based on Gene Ontology (GO) annotation in the six pairwise comparisons among the four groups of mice, i.e., the normal control, the model, the allopurinol, and the noni fruit juice groups.

GO term and gene annotated	Control vs. model	Control vs. noni fruit juice	Control vs. allopurinol	Model vs. noni fruit juice	Model vs. allopurinol	Allopurinol vs. noni fruit juice
Urate biosynthetic process (GO:0034418)						
NM_021463.4 (Prps1)	(2↑,2↓)/(30↑,26↓)	(2↑,5↓)/(80↑,14↓)	(5↑,1↓)/(31↑,31↓)	(0↑,3↓)/(76↑,19↓)	(5↑,1↓)/(29↑,36↓)	(1↑,6↓)/(77↑,14↓)
NM_013632.4 (Pnp)	(0↑,1↓)/(31↑,26↓)	(1↑,2↓)/(77↑,15↓)	(0↑,2↓)/(32↑,33↓)	(1↑,1↓)/(75↑,20↓)	(0↑,1↓)/(33↑,38↓)	(2↑,0↓)/(81↑,16↓)
NM_001164370.1 (Mipoll)	(2↑,1↓)/(20↑,24↓)	(1↑,4↓)/(61↑,15↓)	(1↑,1↓)/(26↑,32↓)	(1↑,3↓)/(63↑,14↓)	(1↑,2↓)/(27↑,30↓)	(1↑,4↓)/(63↑,16↓)
Urate metabolic process (GO:0046415)						
XM_006503685.3 (predicted: facilitated glucose transporter, Slc2a9, transcript variant X3)	(3↑,5↓)/(129↑,127↓)	(3↑,10↓)/(343↑,66↓)	(3↑,6↓)/(129↑,152↓)	(3↑,7↓)/(326↑,73↓)	(2↑,2↓)/(128↑,146↓)	(3↑,6↓)/(352↑,69↓)
XM_006503881.3 (predicted: Gckr, transcript variant X1)	(0↑,0↓)/(13↑,5↓)	(0↑,0↓)/(27↑,4↓)	(0↑,0↓)/(9↑,9↓)	(0↑,0↓)/(25↑,7↓)	(0↑,0↓)/(10↑,15↓)	(0↑,0↓)/(24↑,3↓)
NM_009198.3 (sodium phosphate, Slc7a1, transcript variant 1)	(0↑,1↓)/(7↑,12↓)	(0↑,1↓)/(26↑,7↓)	(1↑,1↓)/(15↑,13↓)	(0↑,0↓)/(29↑,7↓)	(1↑,0↓)/(14↑,8↓)	(0↑,2↓)/(25↑,7↓)
NM_013632.4 (Pnp)	(0↑,1↓)/(31↑,26↓)	(1↑,2↓)/(77↑,15↓)	(0↑,2↓)/(32↑,33↓)	(1↑,1↓)/(75↑,20↓)	(0↑,1↓)/(33↑,38↓)	(2↑,0↓)/(81↑,16↓)
NM_009203.3 (organic anion/cation transporter, Slc22a12)	(0↑,0↓)/(2↑,2↓)	(0↑,0↓)/(4↑,2↓)	(0↑,0↓)/(0↑,4↓)	(0↑,0↓)/(3↑,3↓)	(0↑,0↓)/(0↑,2↓)	(0↑,0↓)/(4↑,2↓)
NM_021463.4 (Prps1)	(2↑,2↓)/(30↑,26↓)	(2↑,5↓)/(80↑,14↓)	(5↑,1↓)/(31↑,31↓)	(0↑,3↓)/(75↑,19↓)	(5↑,1↓)/(29↑,36↓)	(1↑,6↓)/(77↑,14↓)
NM_134069.3 (sodium phosphate, Slc7a3, transcript variant 1)	(2↑,0↓)/(17↑,21↓)	(0↑,2↓)/(59↑,6↓)	(2↑,0↓)/(19↑,25↓)	(0↑,4↓)/(62↑,7↓)	(1↑,1↓)/(15↑,20↓)	(0↑,4↓)/(64↑,5↓)
NM_025807.3 (monocarboxylic acid transporters, Slc16a9)	(3↑,2↓)/(48↑,59↓)	(0↑,4↓)/(136↑,53↓)	(4↑,2↓)/(63↑,83↓)	(0↑,5↓)/(137↑,42↓)	(3↑,1↓)/(66↑,64↓)	(0↑,5↓)/(143↑,38↓)
NM_001164370.1 (Mipoll)	(2↑,1↓)/(20↑,24↓)	(1↑,4↓)/(61↑,15↓)	(1↑,1↓)/(26↑,32↓)	(1↑,3↓)/(63↑,14↓)	(1↑,2↓)/(27↑,30↓)	(1↑,4↓)/(63↑,16↓)
XM_006506148.3 (predicted: TED:ATP-binding cassette, WHITE, Abcg2, transcript variate X1)	(1↑,0↓)/(8↑,21↓)	(0↑,1↓)/(42↑,12↓)	(0↑,1↓)/(18↑,26↓)	(0↑,2↓)/(43↑,5↓)	(0↑,1↓)/(19↑,17↓)	(0↑,1↓)/(46↑,9↓)
NM_008061.4 (glucose-6-phosphatase, G6pc)	(2↑,4↓)/(77↑,80↓)	(2↑,6↓)/(186↑,51↓)	(2↑,6↓)/(64↑,101↓)	(2↑,4↓)/(183↑,52↓)	(2↑,3↓)/(62↑,92↓)	(3↑,2↓)/(210↑,40↓)
Urate transport (GO:0015747)						
NM_134069.3 (sodium phosphate, transcript variant 1)	(2↑,0↓)/(17↑,21↓)	(0↑,2↓)/(59↑,6↓)	(2↑,0↓)/(19↑,25↓)	(0↑,4↓)/(62↑,7↓)	(1↑,1↓)/(15↑,20↓)	(0↑,4↓)/(64↑,5↓)
NM_009203.3 (organic anion/cation, transporter, Slc22a12)	(0↑,0↓)/(2↑,2↓)	(0↑,0↓)/(4↑,2↓)	(0↑,0↓)/(0↑,4↓)	(0↑,0↓)/(3↑,3↓)	(0↑,0↓)/(0↑,2↓)	(0↑,0↓)/(4↑,2↓)
XM_006506148.3 (predicted: TED:ATP-binding cassette, WHITE, Abcg2, transcript variant X1)	(1↑,0↓)/(8↑,21↓)	(0↑,1↓)/(42↑,12↓)	(0↑,1↓)/(18↑,26↓)	(0↑,2↓)/(43↑,5↓)	(0↑,1↓)/(19↑,17↓)	(0↑,1↓)/(46↑,9↓)

Data are presented as number of (known)/(novel) microRNAs. Symbols “↑” and “↓” indicate up-regulation and down-regulation, respectively.

**Table 3:** Expression patterns of target genes and the known differentially expressed microRNAs involved in uric acid production based on Gene Ontology (GO) annotation in the six pairwise comparisons among the four groups of mice, i.e., the normal control, the model, the allopurinol, and the noni fruit juice groups.

GO term and gene annotated	Control vs. model	Control vs. noni fruit juice	Control vs. allopurinol	Model vs. noni fruit juice	Model vs. allopurinol	Allopurinol vs. noni fruit juice
Urate biosynthetic process (GO:0034418)						
NM_021463.4 (Prps1) ↑	let-7e-5p; miR-181b-5p	let-7e-5p; miR-181b-5p	let-7e-5p; miR-33-5p; let-7a-5p; let-7c-5p; let-7b-5p	—	miR-33-5p; let-7e-5p; let-7c-5p; let-7a-5p; let-7b-5p	miR-181b-5p
NM_021463.4 (Prps1) ↓	miR-124-3p; miR-33-5p	miR-124-3p; miR-33-5p; let-7d-5p; let-7c-5p; let-7b-5p	miR-124-3p	let-7d-5p; let-7c-5p; let-7b-5p	miR-181b-5p	miR-33-5p; let-7c-5p; let-7d-5p; let-7a-5p; let-7b-5p; let-7e-5p
NM_013632.4 (PnP) ↑	—	miR-214-3p	—	miR-214-3p	—	miR-140-3p; miR-214-3p
NM_013632.4 (Pnp) ↓	miR-7051-5p	miR-7051-5p; miR-140-3p	miR-140-3p; miR-7051-5p	miR-140-3p	miR-140-3p	—
NM_001164370.1 (Mipoll) ↑	miR-429-3p; miR-205-5p	miR-199a-5p	miR-485-5p	miR-199a-5p	miR-485-5p	miR-199a-5p
NM_001164370.1 (Mipoll) ↓	miR-485-5p	miR-532-5p; miR-429-3p; miR-485-5p; miR-205-5p	miR-429-3p	miR-429-3p; miR-532-5p; miR-205-5p	miR-429-3p; miR-532-5p	miR-532-5p; miR-429-3p; miR-485-5p; miR-205-5p
Urate metabolic process (GO:0046415)						
XM_006503685.3 (predicted: facilitated glucose transporter, Slc2a9, transcript variant X3) ↑	miR-7052-3p; miR-145a-3p; miR-484	miR-199a-5p; miR-214-3p; miR-709	miR-211-5p; miR-328-3p; miR-145a-3p	miR-709; miR-199a-5p; miR-214-3p	miR-211-5p; miR-149-5p	miR-709; miR-214-3p; miR-199a-5p
XM_006503685.3 (predicted: facilitated glucose transporter, Slc2a9, transcript variant X3) ↓	miR-500-3p; miR-709; miR-7051-5p; miR-6914-3p; miR-149-5p	miR-7052-3p; miR-145a-3p; miR-500-3p; miR-7051-5p; miR-6914-3p; miR-204-5p; miR-149-5p; miR-501-3p; miR-484; miR-328-3p	miR-501-3p; miR-7052-3p; miR-500-3p; miR-709; miR-7051-5p; miR-6914-3p	miR-7052-3p; miR-145a-3p; miR-484; miR-501-3p; miR-204-5p; miR-328-3p; miR-149-5p	miR-7052-3p; miR-501-3p	miR-145a-3p; miR-211-5p; miR-149-5p; miR-204-5p; miR-328-3p; miR-484
NM_009198.3 (predicted: Gckr, transcript variant X1) ↑	—	—	miR-382-5p	—	miR-382-5p	—
NM_009198.3 (predicted: Gckr, transcript variant X1) ↓	miR-7a-5p	miR-7a-5p	miR-7a-5p	—	—	miR-382-5p; miR-7a-5p
NM_013632.4 (Pnp) ↑	—	miR-214-3p	—	miR-214-3p	—	miR-140-3p; miR-214-3p
NM_013632.4 (Pnp) ↓	miR-7051-5p	miR-7051-5p; miR-140-3p	miR-140-3p; miR-7051-5p	miR-140-3p	miR-140-3p	—
NM_021463.4 (Prpsl) ↑	let-7e-5p; miR-181b-5p	let-7e-5p; miR-181b-5p	let-7e-5p; miR-33-5p; let-7a-5p; let-7c-5p; let-7b-5p	—	miR-33-5p; let-7e-5p; let-7c-5p; let-7a-5p; let-7b-5p	miR-181b-5p
NM_021463.4 (Prpsl) ↓	miR-124-3p; miR-33-5p	miR-124-3p; miR-33-5p; let-7d-5p; let-7c-5p; let-7b-5p	miR-124-3p	let-7d-5p; let-7c-5p; let-7b-5p	miR-181b-5p	miR-33-5p; let-7c-5p; let-7d-5p; let-7a-5p; let-7b-5p; let-7e-5p
NM_134069.3 (sodium phosphate, Slc17a3, transcript variant 1) ↑	miR-6996-5p; miR-1934-5p	—	miR-1934-5p; miR-211-5p	—	miR-211-5p	—
NM_134069.3 (sodium phosphate, Slc17a3, transcript variant 1) ↓	—	miR-204-5p; miR-122-5p	—	miR-6996-5p; miR-1934-5p; miR-204-5p; miR-122-5p	miR-6996-5p	miR-211-5p; miR-1934-5p; miR-204-5p; miR-122-5p
NM_025807.3 (monocarboxylic acid transporters, Slc16a9) ↑	miR-7117-3p; miR-6948-3p; miR-151-5p	—	miR-330-5p; miR-6948-3p; miR-151-5p; miR-151-3p	—	miR-330-5p; miR-6948-3p; miR-151-5p	—
NM_025807.3 (monocarboxylic acid transporters, Slc16a9) ↓	miR-7051-5p; miR-124-3p	miR-124-3p; miR-7051-5p; miR-151-3p; miR-326-3p	miR-124-3p; miR-7051-5p	miR-7117-3p; miR-6948-3p; miR-151-3p; miR-326-3p; miR-151-5p	miR-7117-3p	miR-330-5p; miR-6948-3p; miR-151-3p; miR-326-3p; miR-151-5p
NM_001164370.1 (Mipoll) ↑	miR-429-3p; miR-205-5p	miR-199a-5p	miR-485-5p	miR-199a-5p	miR-485-5p	miR-199a-5p
NM_001164370.1 (Mipoll) ↓	miR-485-5p	miR-532-5p; miR-429-3p; miR-485-5p; miR-205-5p	miR-429-3p	miR-429-3p; miR-532-5p; miR-205-5p	miR-429-3p; miR-532-5p	miR-532-5p; miR-429-3p; miR-485-5p; miR-205-5p
XM_006506148.3 (predicted: TED:ATP-binding cassette, WHITE, Abcg2, transcript variant X1) ↑	miR-346-5p	—	—	—	—	—
XM_006506148.3 (predicted: TED:ATP-binding cassette, WHITE, Abcg2, transcript variant X1) ↓	—	miR-193b-3p	miR-193b-3p	miR-193b-3p; miR-346-5p	miR-346-5p	miR-193b-3p
NM_008061.4 (glucose-6-phosphatase, G6pc) ↑	miR-671-5p; miR-17-5p	miR-214-3p; miR-709	miR-485-5p; miR-3074-2-3p	miR-709; miR-214-3p	miR-485-5p; miR-3074-2-3p	miR-709; miR-214-3p; miR-93-5p
NM_008061.4 (glucose-6-phosphatase, G6pc) ↓	miR-8116; miR-485-5p; miR-709; miR-574-5p	miR-3074-2-3p; miR-17-5p; miR-8116; miR-93-5p; miR-485-5p; miR-574-5p	miR-17-5p; miR-8116; miR-574-5p; miR-709; miR-470-5p; miR-93-5p	miR-17-5p; miR-3074-2-3p; miR-671-5p; miR-93-5p	miR-17-5p; miR-671-5p; miR-93-5p	miR-3074-2-3p; miR-485-5p
Urate transport (GO:0015747)						
NM_134069.3 (sodium phosphate, Slc17a3, transcript variant 1) ↑	miR-6996-5p; miR-1934-5p	—	miR-1934-5p; miR-211-5p	—	miR-211-5p	—
NM_134069.3 (sodium phosphate, Slc17a3, transcript variant 1) ↓	—	miR-204-5p; miR-122-5p	—	miR-6996-5p; miR-1934-5p; miR-204-5p; miR-122-5p	miR-6996-5p	miR-211-5p; miR-1934-5p; miR-204-5p; miR-122-5p
XM_006506148.3 (predicted: TED:ATP-binding cassette, WHITE, Abcg2, transcript variant X1) ↑	miR-346-5p	—	—	—	—	—
XM_006506148.3 (predicted: TED:ATP-binding cassette, WHITE, Abcg2, transcript variant X1) ↓	—	miR-193b-3p	miR-193b-3p	miR-193b-3p; miR-346-5p	miR-346-5p	miR-193b-3p

Symbols “↑” and “↓” indicate up-regulation and down-regulation, respectively. Symbol “—” indicates miRNAs not detected.

#### 3.2.5. KEGG metabolic pathway enrichment analysis of the target genes of differentially expressed microRNAs

**Figure 5:**
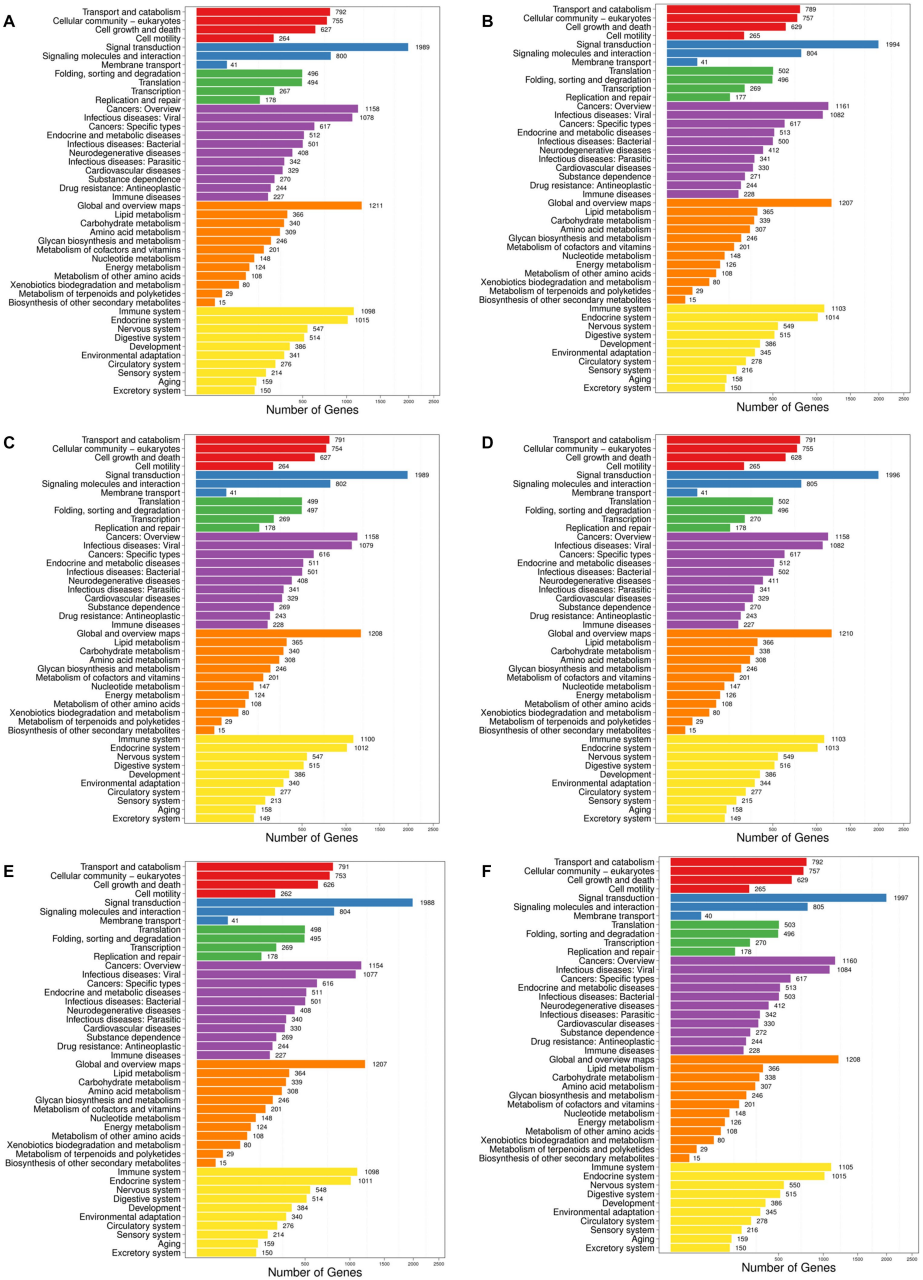
Metabolic pathway enrichment analysis of the target genes of differentially expressed microRNAs based on the Kyoto Encyclopedia of Genes and Genomes (KEGG) database identified in the pairwise comparisons of four groups of mice, including the normal control vs. model groups **(A)**, the normal control vs. noni fruit juice groups **(B)**, the normal control vs. allopurinol groups **(C)**, the model vs. noni fruit juice groups **(D)**, the model vs. allopurinol groups **(E)**, and the allopurinol vs. noni fruit juice groups **(F)**. The six categories of metabolic pathways in KEGG database are shown in six different color blocks, i.e., red, blue, green, purple, orange, and yellow, representing cellular processes, environmental information processing, genetic information processing, human diseases, metabolism, and organismal systems, respectively. Number of genes represents the target genes of the differentially expressed miRNAs.

**Figure 6:**
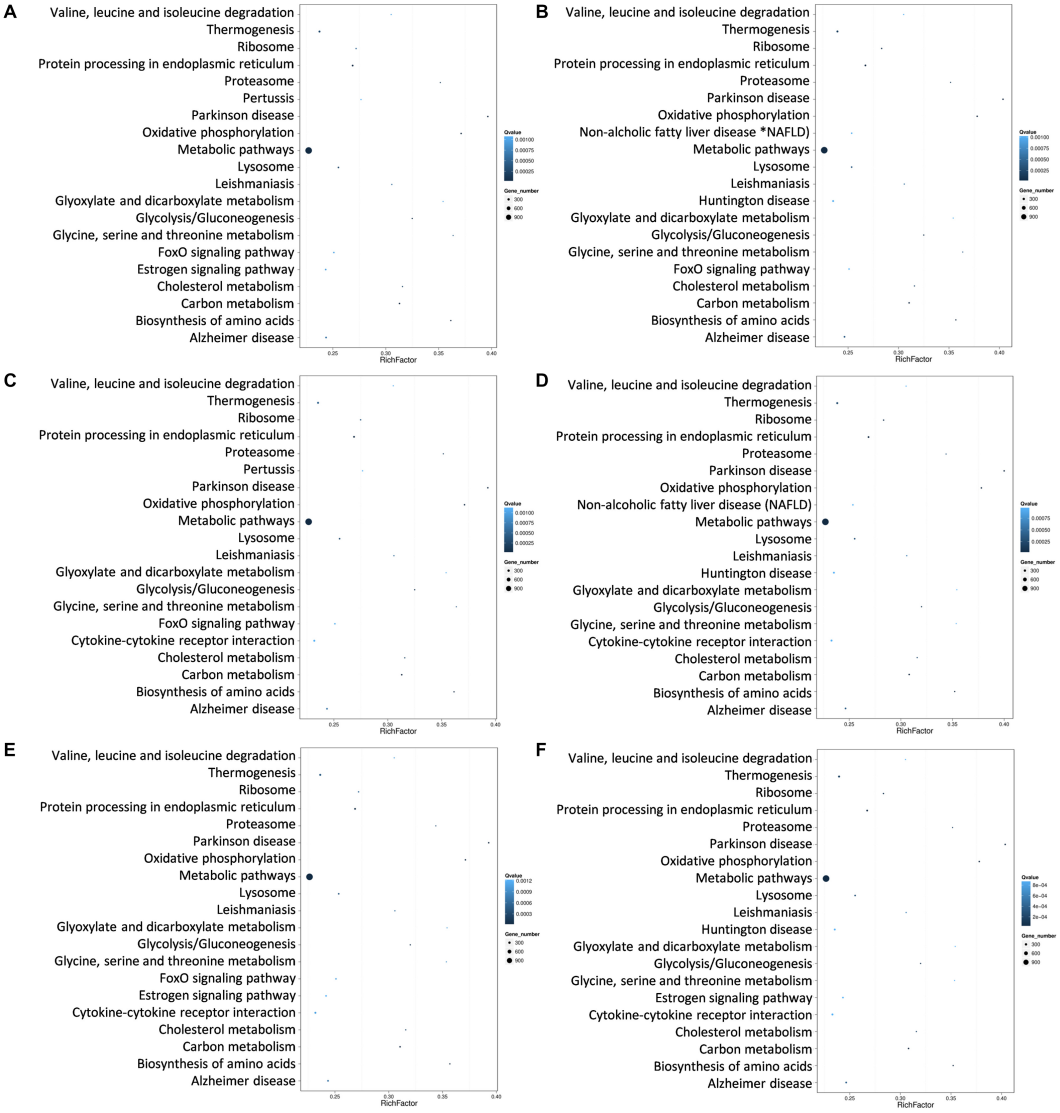
Scatter plots of the top 20 enriched pathway terms based on the Kyoto Encyclopedia of Genes and Genomes (KEGG) database of the target genes of the differentially expressed microRNAs identified in the pairwise comparisons among the four groups of mince, including the normal control vs. model groups **(A)**, the normal control vs. noni fruit juice groups **(B)**, the normal control vs. allopurinol groups **(C)**, the model vs. noni fruit juice groups **(D)**, the model vs. allopurinol groups **(E)**, and the allopurinol vs. noni fruit juice groups **(F)**. The Rich Factor is the ratio of the target gene number annotated in the pathway term to all gene number annotated in the pathway term; the bigger the Rich Factor, the greater the degree of enrichment. The *Q*-value is the corrected *p*-value, ranging from 0 to 1; the lower the *Q*-value, the greater the level of enrichment.

Generally, different genes coordinate with each other to perform their biological functions. In order to comprehensively explore the explicit functions of the target genes of the differentially expressed miRNAs in mice with hyperuricemia, the enrichment analyses of the target genes of the differentially expressed miRNAs in the six pairwise comparisons of the four groups of mice were performed based on the KEGG database to determine the main intracellular signal transduction and metabolic pathways involved in the occurrence of hyperuricemia. The results showed that the target genes of the differentially expressed miRNAs were enriched in six categories of metabolic pathways in the KEGG database, including Cellular Processes, Environmental Information Processing, Genetic Information Processing, Human Diseases, Metabolism, and Organismal Systems (Figure 5). The target genes were highly enriched in the pathways of the Environmental Information Processing, e.g., the Signal transduction, while the “Cancers: Overview” and “Infectious diseases: Viral” were the top two highly enriched pathways in Human Diseases. In the category of Metabolism, the target genes were highly enriched in Global and overview maps. The target genes were highly enriched in both Immune system and Endocrine system of the Organismal Systems. The target genes were relatively less enriched in both categories of Cellular Process and Genetic Information Processing. Among the top 20 most enriched pathways, the target genes of the differentially expressed miRNAs were enriched the most significantly in the metabolic pathways (Figure 6). Subsequently, the regulatory mechanisms of noni fruit juice involved in mice with hyperuricemia were further investigated based on these most enriched metabolic pathways. Future studies are needed to clarify the functions of the target genes enriched in the metabolic pathway involved in the occurrence of hyperuricemia and the role of noni fruit juice in attenuating hyperuricemia in mice.

Because the UA was the final product of the purine metabolism (with XOD playing the rate-limiting role in the terminal step of purine metabolism that converted xanthine to UA) and the results of our biochemical analysis revealed the decreased contents of both serum UA and XOD in mice with hyperuricemia treated with either noni fruit juice or allopurinol, the xanthine dehydrogenase/oxygenase (KEGG orthology K00106) in the KEGG pathway of purine metabolism (KEGG pathway map00230) was further investigated to characterize the genes and differentially expressed miRNAs related to XOD in order to explore the molecular mechanisms regulating the decreased levels of XOD in mice with hyperuricemia (Table 4 and Supplementary Table S11). The results showed that a large number of known and novel miRNAs were involved in the molecular regulation of XOD in the purine metabolism (Table 5 and Supplementary Table S11). Further studies are needed to verify the findings revealed in this study, i.e., the miRNAs regulated the production of XOD and ultimately the decreased level of serum UA in mice with hyperuricemia and the noni fruit juice regulated XOD to play its therapeutic role in the treatment of mice with hyperuricemia.

**Table 4:** Expression patterns of differentially expressed microRNAs in the six pairwise comparisons among the four groups of mice, i.e., the normal control, the model, the allopurinol, and the noni fruit juice groups, with the target genes related to xanthine dehydrogenase/oxygenase involved in the metabolic pathways of hyperuricemia based on the Kyoto Encyclopedia of Genes and Genomes (KEGG) enrichment analysis.

XOD related gene	Control vs. model	Control vs. noni fruit juice	Control vs. allopurinol	Model vs. noni fruit juice	Model vs. allopurinol	Allopurinol vs. noni fruit juice
NM_011723.3 (Xdh)	(6↑,4↓)/(77↑,79↓)	(1↑,10↓)/(223↑,42↓)	(1↑,5↓)/(75↑,94↓)	(1↑,8↓)/(209↑,48↓)	(1↑,5↓)/(80↑,98↓)	(0↑,5↓)/(226↑,35↓)
NM_026670.4 (zinc finger, Zmym1)	(0↑,0↓)/(1↑,3↓)	(0↑,0↓)/(2↑,2↓)	(0↑,0↓)/(0↑,5↓)	(0↑,0↓)/(2↑,1↓)	(0↑,0↓)/(0↑,2↓)	(0↑,0↓)/(3↑,0↓)

Data are presented as number of (known)/(novel) microRNAs. Symbols “↑” and “↓” indicate up-regulation and down-regulation, respectively.

**Table 5:** Known microRNAs of the pairwise comparisons among the four groups of mice, i.e., the normal control, the model, the allopurinol, and the noni fruit juice groups, with the target genes related to xanthine dehydrogenase/oxygenase involved in the metabolic pathways of hyperuricemia based on the Kyoto Encyclopedia of Genes and Genomes (KEGG) enrichment analysis.

XOD related gene	Control vs. model	Control vs. noni fruit juice	Control vs. allopurinol	Model vs. noni fruit juice	Model vs. allopurinol	Allopurinol vs. noni fruit juice
NM_011723.3 (Xdh) ↑	miR-93-3p; miR-346-5p; miR-15a-5p; miR-486b-3p; miR-484; miR-15b-5p	miR-497a-5p	miR-3074-2-3p	miR-497a-5p	miR-3074-2-3p	—
NM_011723.3 (Xdh) ↓	miR-195a-5p; miR-6953-3p; miR-7051-5p; miR-574-5p	miR-486a-3p; miR-3074-2-3p; miR6953-3p; miR-7051-5p; miR-15a-5p; miR-15b-5p; miR-484; miR-195a-5p; miR-574-5p; miR-486b-3p	miR-486a-3p; miR-574-5p; miR-195a-5p; miR-6953-3p; miR-7051-5p	miR-486a-3p; miR-3074-2-3p; miR-346-5p; miR-93-3p; miR-15a-5p; miR-15b-5p; miR-484; miR-486b-3p	miR-486a-3p; miR-346-5p; miR-93-3p; miR-15a-5p; miR-15b-5p	miR-3074-2-3p; miR-15a-5p; miR-484; miR-15b-5p; miR-486b-3p

Symbols “↑” and “↓” indicate up-regulation and down-regulation, respectively. Symbol “—” indicates miRNAs not detected.

## 4. Discussion

In this study, both biochemical and next generation high-throughput RNA-Seq analyses were performed to explore the effect of noni fruit juice on hyperuricemia in mice. The results of biochemical analysis showed that the noni fruit juice could significantly reduce the contents of serum UA and XOD in mouse model of hyperuricemia induced by PO. The RNA-Seq analysis showed that the decrease of the serum UA and XOD levels was probably caused by the inhibited expression of XOD by noni fruit juice (below). The molecular and pharmacological mechanisms underlying the effect of noni fruit juice on hyperuricemia in mice were further investigated based on GO annotation and KEGG enrichment analyses of the target genes of differentially expressed miRNAs.

### 4.1. Variations in the levels of hyperuricemia related biochemical factors caused by the treatment of noni fruit juice

In recent years, studies on hyperuricemia have attracted increasing attention worldwide due to its elevated incidence rate in the populations of young people. However, the effective medicines used in the clinical treatment of hyperuricemia to decrease the blood UA are sparse (42). Therefore, it is urgent to develop novel drugs to reduce the level of blood UA. To date, several animal models of hyperuricemia have been established to develop new drugs for the treatment of hypouricemia. Among these models, PO has been widely used to induce high UA in rodents due to its capability of preventing uricase from degrading UA to generate allantoin (43) as well as its advantages of fast operation and low cost (44). Therefore, the PO was used to induce hyperuricemia in mice in this study. Furthermore, the concentration of serum UA is an important parameter of human health, while hyperuricemia is generally diagnosed based on the abnormally high level of blood UA (45). Therefore, the change of serum UA homeostasis is closely related to hyperuricemia. Our results showed that in 7 days, the serum UA level in mice of the model group treated with PO was significantly higher than that in the normal control group. These results were consistent with those previously reported (27), indicating that the mouse model of hyperuricemia induced by PO was successfully established. Furthermore, the results of biochemical analysis showed that the content of serum UA in mice of hyperuricemia was significantly decreased by the administration of either noni fruit juice or allopurinol, suggesting the alleviation effect of noni fruit juice on hyperuricemia. It was noted that although the UA level was not restored by the treatment of noni fruit juice to the level of the normal control group of mice, the UA levels in both the noni fruit juice and the allopurinol (the positive control) groups were significantly different from that of the model group, suggesting that the noni fruit juice probably altered the production of UA with different mechanisms from that of the allopurinol. Future studies are needed to clarify the molecular mechanisms underlying the alleviation effects of both noni fruit juice and allopurinol on hyperuricemia.

As one of the key enzymes in the production of UA in human, XOD plays the rate-limiting role in the terminal step of purine metabolism that converts hypoxanthine to xanthine, which is used to generate UA (46); as the XOD activity is increased *in vivo*, the purine metabolism is increased, ultimately resulting in the increased UA synthesis. In our study, the biochemical analysis showed that the content of serum XOD in mice of the model group was significantly increased, compared with that of the normal control groups. As one of the effective inhibitors of XOD, allopurinol has been commonly used in the treatment of gout and hyperuricemia for decades (47). As expected, the serum XOD level in mice with hyperuricemia was significantly reduced by the treatment of allopurinol, even below the level of that in the normal control group. Similarly, compared with the model group, the serum XOD level in mice with hyperuricemia was significantly reduced by the treatment of noni fruit juice, suggesting that the noni fruit juice probably functioned to reduce the content of UA by inhibiting the enzymatic activities of XOD, as reported in the previous studies *in vitro* showing that noni fruit juice could inhibit the XOD activity (21). These results were consistent with those previously reported (27), revealing the strong inhibitory effect of both allopurinol and noni fruit juice on XOD.

The UA is mainly excreted by kidney, while insufficient excretion of UA leads to kidney damage (48). Both Cr and BUN are important indicators of renal function (49). In particular, the renal damage is accompanied by the increased levels of serum Cr and BUN, indicating decreased clearance of Cr and urea, respectively (28). Our results were consistent with those reported previously, showing that in comparison to the model group, the contents of serum Cr and BUN in mice with hyperuricemia were significantly reduced by the treatment of either noni fruit juice or allopurinol (*p* < 0.0001), suggesting that the noni fruit juice could promote the excretion of UA without causing deleterious effect on the renal functions of the mice.

### 4.2. MicroRNAs and metabolic pathways involved in the therapeutic treatment of hyperuricemia in mice by noni fruit juice

Hyperuricemia is diagnosed by excessive UA production and/or decreased excretion of UA from mainly kidney and slightly intestine, ultimately leading to excessive increase of serum UA concentration (3). The UA homeostasis in human is a complex and highly hereditary process, including the biosynthesis of metabolic urate, the reabsorption of renal urate, and the excretion of renal and extrarenal urate (50). As the final metabolic product of endogenous and exogenous purines in human, UA is excreted in human, instead of allantoin in some other animals (51, 52). The UA in plasma is filtered out in glomerulus of the kidney and then transported bidirectionally along renal tubules. This process includes renal tubule reabsorption, re-secretion, and reabsorption after secretion. The transport of UA in kidney is mainly accomplished by UA transporters, which act synergistically to maintain the steady level of UA. The abnormality of these transporters could cause UA to be excreted in urine and accumulated in the body, ultimately causing the hyperuricemia.

In order to further explore the pharmacological and molecular mechanisms underlying the effect of noni fruit juice on the therapeutic treatment of hyperuricemia in mice, the differentially expressed miRNAs and their target genes were identified in mice using the next generation high-throughput RNA-Seq technology. The target genes were further annotated by the GO database and enriched by the KEGG database to reveal the metabolic pathways involved in the pharmacological and molecular mechanisms regulating the therapeutic treatment of hyperuricemia in mice by noni fruit juice.

To date, a few dozens of miRNAs involved in hyperuricemia have been identified (22, 53–56) with many of them revealed in our study, showing various expression patterns largely consistent with those previously reported. For example, studies have shown that the expression of miR-143-3p is significantly decreased in mice with hyperuricemia to cause the increased activity of the glucose and fructose transporter GLUT9 (57), while our results showed that the miR-143-3p was significantly increased by the treatment of noni fruit juice. Furthermore, the down-regulation of miR-92a is involved in the KLF2-VGEFA axis and the angiogenesis in hyperuricemia (58), while our results revealed significantly increased expression of miR-92a by the treatment of noni fruit juice. The expression of miR-9 is increased by epigallocatechin gallate (EGCG) to regulate the NF-κB and JAK–STAT pathways in NRK-49F cells (59). Similarly, our results showed that the expression of miR-9 was significantly increased by the treatment of noni fruit juice. It was worthy of noting that the expression patterns of some of these miRNAs were different between the treatments of noni fruit juice and allopurinol, suggesting the varied molecular mechanisms regulating the alleviation effects of noni fruit juice and allopurinol. For example, the expression of miR-181a, which is closely related to the production of UA and renal damage in the chronic kidney disease by the down-regulation of the TLR/NF-κB pathway (60), was significantly up-regulated by the treatment of allopurinol but down-regulated by the treatment of noni fruit juice in our study, whereas the expressions of several miRNAs (e.g., miR-451a, miR-155-5p, and miR-149-5p) were significantly down-regulated by PO, noni fruit juice, and allopurinol, suggesting the varied functions of PO, noni fruit juice, and allopurinol in the development and treatment of hyperuricemia.

The GO annotation results showed that a group of target genes of differentially expressed miRNAs were annotated in urate biosynthetic process, urate metabolic process, and urate transport related to the etiology of hyperuricemia with the known and novel miRNAs identified in the pairwise comparisons of the four groups of mice. Further studies are needed to identify the explicit functions of these novel miRNAs in the treatment of hyperuricemia in mice, while some of the known miRNAs are discussed here due to their functions related to hyperuricemia as previously reported. For example, the miR-214-3p was highly expressed in mice of the noni fruit juice group. Studies have shown that the level of serum miR-214 in patients with hyperuricemia is lower than that in healthy controls, showing negative correlation with the level of UA (56). These results suggested that the noni fruit juice was probably involved in the urate biosynthetic process and urate metabolic process by up-regulating the expression of miR-214-3p, ultimately decreasing the level of serum UA in mice with hyperuricemia. Furthermore, the miR-17-5p was significantly up-regulated in the model group of mice. These results were consistent with those previously reported, showing that hsa-mir-17-5p was significantly up-regulated in plasma of patients with hyperuricemia and gout compared with normal subjects (*p* < 0.001) (53). Moreover, the miR-17-5p was significantly down-regulated in both the noni fruit juice and the allopurinol groups, suggesting that the noni fruit juice and allopurinol could reduce the expression of miR-17-5p. Therefore, these results indicated that both noni fruit juice and allopurinol reduced the level of serum UA in mice with hyperuricemia, probably by inhibiting the expression of miR-17-5p, which was likely involved in the metabolic pathways of urate biosynthetic process, urate metabolic process, and urate transport. Further studies are necessary to explicitly identify the regulatory functions of miR-17-5p and underlying molecular and pharmacological mechanisms in the metabolic pathways of urate biosynthetic process, urate metabolic process, and urate transport in mice with hyperuricemia. In addition, previous microarray studies revealed the down-regulation of miR-149-5p by the treatment of allopurinol in mice, while the miR-149-5p expression was significantly up-regulated in UA-stimulated hepatocytes (61). Furthermore, as one of the target genes of miR-149-5p, the *fibroblast growth factor 21* (*FGF21*) expression was inhibited by the overexpression of miR-149-5p, while the UA-induced lipid deposition was decreased in the hepatocytes, indicating that the expression of miR-149-5p was significantly up-regulated by UA in hepatocytes to increase the lipid accumulation in hepatocytes via the interaction between miR-149-5p and FGF21 (61). These results were consistent with the findings revealed in our study, showing that the expression of miR-149-5p was differentially altered in mice of both noni fruit juice and model groups and involved in the urate metabolic process. Future studies are need to explore the explicit functions of miR-149-5p involved in the pathogenesis of hyperuricemia.

The KEGG enrichment analysis of the target genes of the differentially expressed miRNAs in the pairwise comparisons among the four groups of mice identified the significantly enriched metabolic pathways involved in the development and treatment of hyperuricemia. Future studies are needed to further explore the functions of these metabolic pathways and miRNAs enriched in the therapeutic effects of noni fruit juice on hyperuricemia. For example, as one of the key enzymes involved in the biosynthesis of UA in the purine metabolism, the XOD, also known as xanthine oxidoreductase or XOR, is coded by the xanthine dehydrogenase gene *XDH* (62). Therefore, the XOD related genes involved in the purine metabolism were further evaluated based on KEGG database to identify the known and novel differentially expressed miRNAs in the pairwise comparisons among the four groups of mice. However, the relationships between these miRNAs and hyperuricemia are rarely reported in the literature. Further studies are needed to identify the explicit functions of these miRNAs involved in hyperuricemia. For example, our results showed that the miR-93-3p was significantly up-regulated in the model group and significantly down-regulated in both the noni fruit juice and allopurinol groups. Studies have shown that miR-93-3p is one of the 10 diagnostic biomarkers in patients with acute kidney injury in the intensive care units (63), suggesting that the increased expression of miR-93-3p in the model group in our study could be related to the renal injury in mice of the model group and could be potentially used as the diagnostic biomarker of renal injury in mice. Future studies are needed to explore the regulatory functions of these miRNAs involved in the therapeutic treatment of hyperuricemia in mice by noni fruit juice and allopurinol. For example, the results of the TargetScan revealed that as the target gene of miR-214-3p, *SLC22A12* encodes the glucose and fruticose transporter URAT1 (22, 59), which functions as the exchanger of UA and anion, ultimately altering the serum UA level through UA reabsorption in human kidney (64, 65). Our results showed the expression of miR-214-3p was significantly increased by the treatment of noni fruit juice. Therefore, the URAT1 has become an important and potential target for the treatment of hyperuricemia (66), and future studies are needed to identify the regulatory functions of miR-214-3p in the synthesis of URAT1 and its effect on the absorption of UA and the treatment of hyperuricemia in mice.

Studies have shown that some miRNAs are involved in both hyperuricemia and gout (22, 67). For example, the expression of miR-146a was significantly increased in patients with gout and involved in both NLRP3 and MyD88/NF-κB pathways (68). Similar results were revealed in our study, showing that the expression of miR-146a-5p was significantly increased in the model group and significantly decreased in both noni fruit juice and allopurinol groups, suggesting the therapeutic effect of noni fruit juice on hyperuricemia and its involvement in the NLRP3 and MyD88/NF-κB pathways. Furthermore, studies have shown that the expression of miR-223-3p is significantly down-regulated in the mouse model of pouch synovium with the expression of NLRP3 inhibited by the overexpression of miR-223-3p, ultimately alleviating the inflammatory effect of gout (69). Our results showed that the expression of miR-223-3p was significantly decreased in both noni fruit juice and allopurinol groups and significantly increased in the model group, suggesting the potential participation of miR-223-3p in the NLRP3 pathway. Moreover, studies have shown that the expression of miR-155 is increased in the gouty arthritis model with the expression level of SHIP-l inhibited and production of proinflammatory cytokines enhanced (70). Our results showed that the expression of miR-155-5p was significantly increased in the model group, suggesting the important roles that miR-155-5p played in the occurrence of hyperuricemia in mice. In addition, a group of miRNAs have been identified as biomarkers of hyperuricemia (53) with some of them revealed in our study with significant regulations in their expressions and correlations with the contents of serum Cr, including miR-17-5p, miR-18a-5p, miR-223-3p, miR-146a-5p, and miR-155-5p, in the noni fruit juice, model, and allopurinol groups in our study. Future studies are necessary to identify the explicit functions of these miRNA involved in the various metabolic pathways related to hyperuricemia.

To date, numerous studies have shown that the natural products, i.e., traditional Chinese medicines, have been revealed with alleviation effect on hyperuricemia, in particular, altering the content of hyperuricemia related biochemical factors (e.g., serum UA, Cr, and BUN) and inhibiting the XOD activities (26, 29, 71–79). For example, an empirical formula Xie-Zhuo-Chu-Bi-Fang is used to treat mice with hyperuricemia, significantly decreasing the content of serum UA, down-regulating the expression of URAT1, and up-regulating miR-34a (26). Furthermore, as a hydrogenated derivative of berberine, dihydroberberine is revealed with effective inhibition of XOD, significantly decreasing the contents of serum Cr and BUN and down-regulating the renal mRNA and protein expression of XOD as well as several other hyperuricemia related biochemical factors in mice with hyperuricemia (29). Together with the findings revealed in our study, these natural products provide a wide spectrum of potentially promising therapeutic treatments of hyperuricemia. Studies have shown the various types of polysaccharides are involved in the treatment and prevention of hyperuricemia, suggesting that the noni fruit juice probably contained the same or similar chemical compositions as the polysaccharides as previously reported (80). Further studies are necessary to identify these functional substances in noni fruit juice (81).

## 5. Conclusion

In this study, we investigated the therapeutic effect of noni fruit juice on mice with hyperuricemia using both biochemical and RNA-Seq analyses and characterized the variations in the contents of hyperuricemia related biochemical factors and differentially expressed miRNAs involved in the molecular and pharmacological mechanisms regulating the therapeutic treatment of hyperuricemia in mice. The results showed that the treatment of noni fruit juice caused significant decrease in the serum UA levels and the contents of XOD, Cr, and BUN in mice with hyperuricemia. The results of RNA-Seq analysis revealed a group of differentially expressed miRNAs involved in the pathogenesis of hyperuricemia in mice. The functional annotation and enrichment analysis of the target genes of differentially expressed miRNAs were performed based on GO and KEGG databases. Our study provided strong experimental evidence to support the therapeutic and pharmacological effects of noni fruit juice on the treatment of hyperuricemia in mice and to support the agricultural and nutritional development of noni plants due to their potential clinical significance in the treatment of hyperuricemia.

## Data availability statement

The original contributions presented in the study are publicly available. This data can be found here: https://ncbi.nlm.nih.gov/bioproject/PRJNA910471.

## Ethics statement

The animal study was reviewed and approved by the Ethics Committee of Jilin University with the approval # 2018SY0602.

## Author contributions

YL, XJL, FS, HL, and ZDL: conceptualization, formal analysis, and writing—original draft preparation. YL, XJL, HL, and ZDL: methodology. MW: software. CC: validation. XHL: investigation. YS: resources. ZYL: data curation. FS, HL, and ZDL: writing—review and editing and supervision. YY: visualization. HL and ZDL: project administration. XJL, HL, and ZDL: funding acquisition. All authors have read and agreed to the published version of the manuscript.

## Funding

This research was funded by Department of Science and Technology of Jilin Province, China (20210202059NC), Department of Education of Jilin Province, China (JJKH20220194KJ), Health Science and Technology Capability Improvement Project of Jilin Province, China (2022JC010), and the PhD Research Project of Jilin Engineering Normal University (BSKJ201923). The APC was funded by HL.

## Conflict of interest

ZYL was employed by Qingdao Haoda Marine Biotechnology Co., Ltd.

The remaining authors declare that the research was conducted in the absence of any commercial or financial relationships that could be construed as a potential conflict of interest.

## Publisher’s note

All claims expressed in this article are solely those of the authors and do not necessarily represent those of their affiliated organizations, or those of the publisher, the editors and the reviewers. Any product that may be evaluated in this article, or claim that may be made by its manufacturer, is not guaranteed or endorsed by the publisher.

## Footnotes

^1^http://geneontology.org/; accessed on 15 March 2022.

^2^https://www.genome.jp/kegg/; accessed on 15 March 2022.

^3^http://www.yeastgenome.org/help/analyze/go-term-finder/; accessed on 15 March 2022.

^4^http://www.ncbi.nlm.nih.gov/sra/; accessed on 09 December 2022.

## Supplementary material

SUPPLEMENTARY TABLE 1 Lists of predicted known and novel microRNAs in the four groups of mice.

SUPPLEMENTARY TABLE 2 List of differentially expressed microRNAs based on the six pairwise comparisons among the four groups of mice.

SUPPLEMENTARY TABLE 3 Target gene intersection of differentially expressed microRNA predicted by TargetScan and miRanda in the pairwise comparisons of the four groups of mice.

SUPPLEMENTARY TABLE 4 Target genes identified by both TargetScan and miRanda of the differentially expressed microRNAs of the normal control and model groups of mice.

SUPPLEMENTARY TABLE 5 Target genes identified by both TargetScan and miRanda of the differentially expressed microRNAs of the normal control and noni fruit juice groups of mice.

SUPPLEMENTARY TABLE 6 Target genes identified by both TargetScan and miRanda of the differentially expressed microRNAs of the normal control and allopurinol groups of mice.

SUPPLEMENTARY TABLE 7 Target genes identified by both TargetScan and miRanda of the differentially expressed microRNAs of the model and the noni fruit juice groups of mice.

SUPPLEMENTARY TABLE 8 Target genes identified by both TargetScan and miRanda of the differentially expressed microRNAs of the model and allopurinol groups of mice.

SUPPLEMENTARY TABLE 9 Target genes identified by both TargetScan and miRanda of the differentially expressed microRNAs of the allopurinol and noni fruit juice groups of mice.

SUPPLEMENTARY TABLE 10 List and expression patterns of microRNAs with the target genes involved in urate biosynthetic process, urate metabolic process, and urate transport, as well as the positive and negative regulations of urate biosynthetic process, urate metabolic process, and urate transport assembly based on the Gene Ontology (GO) annotation in the six pairwise comparisons of the four groups of mice.

SUPPLEMENTARY TABLE 11 List and expression patterns of both known and novel microRNAs targeting xanthine oxidase (XOD) in purine metabolism pathways related to hyperuricemia based on the Kyoto Encyclopedia of Genes and Genomes (KEGG) enrichment analysis in the six pairwise comparisons of the four groups of mice.
